# The Oregon health system transformation: preliminary report of Coordinated Care Organizations in the first year implementation

**DOI:** 10.1186/1472-6963-14-S2-P18

**Published:** 2014-07-07

**Authors:** Carlos Crespo, Ellen Smit

**Affiliations:** 1 Oregon Health Policy Board, Salem, Oregon, USA; 2 Oregon State University, Corvallis, Oregon, USA; 3 Portland State University, Portland, Oregon, USA

## Background

Because of the US Affordable Care Act, 16% of Oregonians without health insurance will be able to obtain coverage through Coordinated Care Organizations (CCO).

## Materials and methods

CCO is a network of all types of health care providers who have agreed to work together in their local communities to serve people who receive health care coverage under the Oregon Health Plan. CCOs are accountable for health outcomes of the population they serve and have one budget that grows at a fixed rate (2%) for mental, physical and ultimately dental care. CCOs are focused on prevention and helping people manage chronic conditions, like diabetes to reduce unnecessary emergency room visit. By using quality, access and financial metrics together, the state can determine whether CCOs are effectively and adequately improving care.

## Results

Emergency department visits by people served by CCOs have decreased 13% since 2011 baseline data.

CCOs reduced hospital admissions for congestive heart failure by 32%, chronic obstructive pulmonary disease by 36% and adult asthma by 18%. Spending for primary care is up by more than 18%. Enrollment in patient-centered primary care homes also increased by 51% since 2012. The percentage of adult patients with diabetes who received at least one A1c blood sugar test in 2013 (74.9%) is down when compared with 2011 baseline (78.5%).

## Conclusion

While some progress has been observed additional improvement in preventive care is expected.

